# A novel therapeutic effect of mannitol-rich extract from the brown seaweed *Sargassum ilicifolium* using in vitro and in vivo models

**DOI:** 10.1186/s12906-023-03840-0

**Published:** 2023-01-31

**Authors:** Amal D Premarathna, Rando Tuvikene, MNR Somasiri, MLWP De Silva, Ranjith Adhikari, TH Ranahewa, RRMKK Wijesundara, SK Wijesekera, IPGHU Dissanayake, Phurpa Wangchuk, Vitalijs Rjabovs, Anura P Jayasooriya, RPVJ Rajapakse

**Affiliations:** 1 Department of Veterinary Pathobiology, Faculty of Veterinary Medicine and Animal Science, University of Peradeniya, Peradeniya, Sri Lanka; 2 School of Natural Sciences and Health, Tallinn University, Narva mnt 29, 10120 Tallinn, Estonia; 3 South Asian Clinical Toxicology Research Collaboration. Faculty of Medicine, National Serpentarium, University of Peradeniya, Peradeniya, Sri Lanka; 4 Department of Zoology, Faculty of Natural Sciences, Open University, Kandy Regional Center, Polgolla, Sri Lanka; 5 Department of Basic Veterinary Sciences, Faculty of Veterinary Medicine and Animal Science, University of Peradeniya, Peradeniya, Sri Lanka; 6 Centre for Molecular Therapeutics, Australian Institute of Tropical health and Medicine, James Cook University, Smithfield, QLD 4878 Australia; 7 National Institute of Chemical Physics and Biophysics, Akadeemia tee 23, 12618 Tallinn, Estonia

**Keywords:** *Sargassum ilicifolium*, D-mannitol, HDF, HaCaT, RAW 264.7, Toxicity, Proliferation, Migration, Rabbit, Wound healing, Anti-inflammatory

## Abstract

**Background:**

Wound healing is an active, complex, integrated series of cellular, physiological, and biochemical changes initiated by the stimulus of injury in a tissue. The present study was performed to investigate the potential wound healing abilities of *Sargassum ilicifolium* crude extracts (CE) that were characterized by ^1^H NMR and FTIR Spectrometric measurements.

**Materials and methods:**

Seaweed samples were collected from southern coastal sites of Sri Lanka. To determine the cytotoxicity and proliferation of *S. ilicifolium* CE were used for the MTT and alamarBlue assays respectively. The scratch and exclusion wound models were used to HaCaT and HDF cells to assess the cell proliferation and migration. RAW 264.7 cells (macrophages) were used to evaluate Nitric Oxide (NO) production and phagocytosis activities. Moreover, Fifteen, 8-week-old, female, New Zealand rabbits were selected and divided into five groups: excision skin wounds (10.40 ± 0.60 mm) were induced in groups I, II, and III. Rabbits in groups I and IV were given *S. ilicifolium* CE (orally, 100 mg/kg day, two weeks), whereas groups II and V were given equal amounts of distilled water. Wound healing properties were measured and wound tissue samples were collated, formalin-fixed, wax-embedded, stained (Hematoxylin and Eosin; Van Gieson) and examined for the healing process.

**Results:**

Anti-inflammatory and wound healing activities were observed in RAW 264.7, HDF and HaCaT cells treated with *S. ilicifolium* aqueous extracts when compared to the control groups*. S. ilicifolium* extracts concentration 8 - 4 μg/μL, (*P<0.05*) had remarkable the highest proliferative and migratory effects on RAW 264.7, HDF and HaCaT cells when compared with the control. RAW 264.7 cell proliferation and/or migration were higher in *S. ilicifolium* extracts (4 μg/μL, 232.8 ± 10.07%) compared with the control (100 %). Scratch wound healing were remarkably enhanced in 24 h, 48 h (*P*<0.05) when treated with *S. ilicifolium* on HaCaT cells. Rabbits treated with the CE of *S. ilicifolium* showed a significantly increased wound healing activities (*P<0.05*) within three days with a close wound area of 57.21 ± 0.77 % compared with control group (26.63 ± 1.09 %). Histopathology, aspartate aminotransferase and alanine aminotransferase levels evidenced no toxic effects on seaweed treated groups. Histopathological results also revealed that the healing process was significantly faster in the rabbit groups which were as treated with CE of *S. ilicifolium* orally with the evidence of enhanced early granulation tissue (connective tissue and angiogenesis) and significant epithelization compared to the control.

**Conclusions:**

Cell proliferation and migration are significantly faster when treated with *S. ilicifolium* aqueous extracts. Moreover, there are no toxic effect of *S. ilicifolium* aqueous extracts on RAW 264.7, HDF and HaCaT cell lines. In this study, it is revealed that *S. ilicifolium* has potential remedial agent; D-Mannitol for skin wound healing properties that by promote keratinocyte and fibroblast proliferation and migration. These findings show that *S. ilicifolium* have promising wound healing properties.

## Introduction

A wound is known as an injury occurs to a part of the body due to an accidental damage or a surgical procedure, especially one in which a skin breakage is observed [1, 2]. Wounds include delayed acute wounds and chronic wounds, which regularly enter a state of pathologic inflammation as a result of a postponed, incomplete, or impaired healing process. Most chronic wounds are ulcers that are linked with diabetes mellitus, venous stasis diseases, ischemia or pressure [3]. The non-healing wounds affect about 3 to 6 million people in the United States resulting in enormous health care expenditures, with the total cost estimated at more than US $3 billion per year [4, 5]. Chronic wounds are also a significant health care problem in Australia, affecting more than half a million patients a day and the treatment costing more than AUD 3.5 billion per year [6]. Ulcers secondary to peripheral vascular disease and diabetes mellitus are a major problem particularly in the aging population. In Sri Lanka, wound infections are rampant and it is one of the major health burden especially the chronic wounds [7]. The chronicity of these wounds increases the risk of bacterial colonization and infection, hindering tissue healing which in most cases, eventually leads to amputations or life-threatening infection [6]. It provides an entry point for systemic infections [8]. As such, proper wound care as well as timely treatment of infections is vital.

Wound healing is a specific process of regeneration of the injured connective tissue of wounds leading to the restoration of injured tissues [9, 10]. It is evident that the chronic wound is prevalent in 4.5 per 1000 life quality by the currently available treatments in geriatric population [11] as well as cutaneous wound are frequent in diabetic’s patients [12, 13]. Wound dressings loaded with antimicrobial agents have gathered much attention in recent times as an avenue for preventing and treating wound infections [14]. Common antibacterial dressings that have shown benefits in wound healing contain silver compounds such as silver nitrate and silver sulfadiazine, however silver-containing products may cause tissue toxicity [15]. Given the diminishing efficacy of currently used topical wound healing agents against many skin pathogens such *Methicillin Resistant Staphylococcus aureus* (MRSA)-inflicted wounds [16], new topical wound healing regimens are much needed. A topical treatment with no significant harmful effect on fibroblast proliferation and wound healing can reduce bacterial burden and lead towards better control of inflammation, wound malodor and purulent secretions.

Although there has been an enormous development in the pharmaceutical drug industry, wound healing drugs are not still satisfactory because of their low availability, high cost, and various detrimental side effects [17]. Therefore, natural products-based drugs derived from medicinal plants and marine natural products are in demand in many developing countries that are rich in herbal traditions due to a common belief that they are safe, reliable, clinically effective, low cost, globally competitive and better tolerated by patients [18, 19]. Natural products would be also useful as therapeutic agent for prevention of wound infections [20]. However, marine natural products including seaweeds and seagrass remain less explored for medicinal applications especially in treating wounds. It is likely that these species are producing diverse secondary metabolites with novel chemical structures, which can be easily manipulated and developed as novel wound-healing drug candidates. There is an enormity of biodiversity within the Sri Lankan marine flora, some of them used in ethnomedicines which remains relatively undiscovered.

Seaweeds are used as functional foods and medicinal herbs in Asian countries [21]. A variety of biological activities are attributed to seaweeds including neuroprotection, anti-tumor, anti-cancer, antioxidant, anti-obesity, anti-inflammatory, and anti-microbial and other biological activities [22]. Biologically active compounds of these plants/marine flora include tannins, triterpenoids, and alkaloids and these chemicals have been found to affect one or more phases of wound healing process [17]. Alkaloids in particular are known to possess exciting biological activities including antibacterial, anti-inflammatory and anti-Alzheime’s disease [23]. Although there are a large number of studies conducted to investigate wound healing activity of different types of terrestrial plant extracts, there is inadequate data on seaweeds.

Herbal preparations thereof have been used to accelerate wound healing since ancient times [24]. The use of these substances is often based only on tradition, without any scientific evidence of their efficacy and with little understanding of the mechanism of action of putative active compounds [25]. The brown seaweed *S. ilicifolium* is reported to exhibit several biological properties that are beneficial to human health, such as anticancer, antimicrobial, anti-inflammatory, and anti-diabetic properties [26, 27]. In vitro study suggests that *S. ilicifolium* stimulates fibroblast migration and proliferation to heal skin wounds [28]. Furthermore, in vivo studies as well as in vitro studies have demonstrated the immunomodulatory properties of *S. ilicifolium*. Specially antioxidant-mediated mechanisms activate the immune system to enhance the host's defense [29]. Using streptozotocin-induced diabetic mice, alginate extract from *S. ilicifolium* was found to be effective in re-epithelializing wound areas, increasing neutrophils, macrophages, fibroblasts, and fibrocytes, as well as collagen density [30]. Previous research study showed, aqueous extracts of *S. ilicifolium* showed no toxic effects on mice [31]. An in vitro and in vivo study found that aqueous extracts of *S. ilicifolium* have better healing effects than control groups [32], but no detailed study seems to have been conducted. The objective of this study was to determine wound healing of *S. ilicifolium* extracts by using rabbit models, and the wound healing evidence was supported by hematological and histopathological data. However, the active compound in this study was reported. *S. ilicifolium* wound healing activity was evaluated using an in vivo model using more precise methods for evaluation. Our previous in vitro study on *S. ilicifolium* extracts using scratch wound healing assay on the L929 cells, showed high rate of cell proliferation and migration. Guided by this preliminary data, in this study, we have investigated the wound healing properties of *S. ilicifolium* extracts using in vitro and in vivo wound-repair model and highlighted its potential therapeutic applications.

## Materials and Methods

### Seaweed material, extract preparation and analysis

#### Seaweed material

Fresh brown seaweed, *S. ilicifolium* was collected from the southern coastal algae beds at Ahangama, in Sri Lanka [33]. The collected brown seaweed sample was authenticated at the “National Herbarium of Peradeniya Botanical Garden” and a voucher specimen of *S. ilicifolium* (Specimen number; SW23/B7) was deposited for future application. Fresh seaweeds were washed thoroughly with tap water, followed by washing with seawater on site to remove all sand particles, impurities, and epiphytes. Finally, the purified seaweeds were washed with distilled water prior to extracts preparation.

### Crude extract (CE) preparation

*S. ilicifolium* sample was dried to remove water, at 40 °C for four days until a constant weight obtained. Then, it was ground with an electric grinder (Herbal Grinder CS-700, China) to obtain 0.5 mm particle size powder and stored at -20 ºC. Then CE preparation was conducted using modified method of Premarathna et al., 2019 [28]. The seaweed powder (100 g) was mixed with 500 mL distilled water and was kept for 1 h at 40 ºC in an ultrasound sonicator (Branson 2510, Danbury, USA) to soak and permit full extraction of bioactive compounds of *S. ilicifolium* into aqueous medium. Then, the soaked seaweed sample was shaken in a tube roller mixer (Denley-spiramix 5, UK) at room temperature. Followed by three days of soaking the preparation was filtered by using a nylon mesh (0.50 μm) to collect CE of *S. ilicifolium*. Finally, the extract was kept in the refrigerator at 4 ºC in a sealed container prior to use the in vivo experiments. Then CE was centrifuged for 10 min at 8000 rpm. After that, the supernatant was filter sterilized through a 0.2 μm filter and used for In vitro study.

### Characterization of algal crude extracts

#### The Fourier Transform Infrared Spectrometer (FTIR) analysis

The FTIR spectra of extracts of *S. illicilolum* material were recorded using the iS50 (Nicolet) Fourier infrared spectrophotometer (Thermo Fisher Scientific, Waltham, MA, USA). The spectra were scanned at room temperature in absorption mode in the wavelength of 400 - 4000 cm^-^^1^, with the Omnic software version 9.2.

### Nuclear magnetic resonance (NMR) spectroscopy

NMR samples were prepared by dissolving the sonicated extract material from *S. illicilolum* (1.5%) and commercially available D-mannitol (98%, Sigma-Aldrich) in D_2_O containing 10mM sodium trimethylsilylpropanesulfonate (DSS) as the internal standard. NMR spectra were recorded on Agilent DD500 NMR spectrometer operating at 500 MHz proton resonance frequency. For ^1^H spectra, 32 transients utilizing 90° pulse, 3 s acquisition time, and 25 s relaxation delay were acquired. Samples were analyzed at 25 °C and the chemical shift was referenced to DSS signal at 0 ppm.

### In vitro assays

#### Cell culture

Human Dermal Fibroblast (HDF, Catalog 106-05a: purchased from Cell Applications, Inc.), RAW 264.7 and Human Epidermal Keratinocyte line (HaCaT, purchased from ATCC) cells were maintained in Dulbecco’s Modified Eagle’s medium (DMEM) supplemented with 10% (v/v) heat-inactivated fetal bovine serum (FBS, Sigma-Aldrich) and 1% penicillin/streptomycin. Cell cultures were maintained at 37 °C in a humidified 5% CO_2_ incubator atmosphere.

### Cell viability on HDF, RAW 264.7 and HaCaT

HDF, RAW 264.7 and HaCaT cells were seeded onto 96-well plates (Corning Glasswork, Corning, NY) at a concentration of 2 x 10^4^ cells/well followed by the addition of CE extracts (ten separate concentrations between 16 μg/μL and 0.02 μg/μL) prepared in cell culture media. A two-fold dilution series were used and eight replicates in each concentration. Negative and positive control tests were also prepared using Milli-Q water and 70% ethanol, respectively. Then contents were removed after 24 h and washed by PBS. 10 µL of MTT (5 mg/mL in PBS) solution and 90 µL DMEM culture media were added and further incubated for 4 h at 37 °C. Next, MTT solution was removed and 100 µL DMSO (dimethyl sulfoxide, spectrophotometric grade) was added. Finally, absorbance was measured using OPTIMA microplate reader (FLUOstar, UK) at the wave lengths of 540 nm. All experiments were triplicated.

### Cell proliferation

In order to determine cell proliferation in the presence of CE extracts on HDF, RAW 264.7 and HaCaT cell lines, alamarBlue assay was conducted as described [34]. Cells were grown in DMEM supplemented with 10% FBS to a final cell density of 2 × 10^4^ cells/well and plates were incubated for approximately at 37 °C for 24 h with 5% CO_2_. Extracts were two-fold serial diluted (16 μg/μL to 0.02 μg/μL) into supplemented media using a separate 96-well plate, applied to the cells, and incubated for different time interval (24 and 48 h) at 37 °C with 5% CO_2_ and examined and photographed periodically. Next, 10 μL alamarBlue regent and 90 μL culture media were mixed and directly added to the wells. Following 4 h incubation, the experimental results were collected using the OPTIMA microplate reader (FLUOstar, UK) at 540 nm. The growth rates were compared to the growth of the negative and positive control.

### Scratch wound healing assay

Cell migration of CE extracts on cells were determine by method of Premarathna et al. 2020. [35] HaCaT cells were seeded (1 × 10^5^ cells/well) into a 48-well tissue culture plate and incubated at 37 °C with 5% CO_2_ until 90% confluent. Cell layer was scratched with a sterile yellow pipette tip (200 μL) across the center of the well and were washed with PBS to remove the debris/detached cells. Then, DMEM medium (150 µL) and CE extracts (150 μL, 0.02 μg/μL) were added to each well photographed and scratch wound closure analyzed (Carl Zeiss Microscopy GmbH software) during time interval at 0, 24 and 48 h.

### Anti-inflammatory activity

Anti-inflammatory activity of *S. illicilolum* extract was observed through nitric oxide (NO) inhibition level in RAW 264.7 cells. Cells were firstly seeded at a density of 1 × 10^6^ cells/well in a 96-microwell plate overnight in a humidified atmosphere (CO_2_ 5%, 37 °C). Subsequently, cells were treated by *S. illicilolum* extract at 4 µg/µL concentrations and incubated at 37 °C for 24 h. Then *S. illicilolum* extract was discarded from 96 well plate. Further this plate was washed with phosphate buffer saline (PBS) to remove test solution. Consequently, NO assay was performed according to the manufacture protocol by using nitrite assay kit (Griess reagent, Sigma-aldrich). Accordingly, the amount of accumulated nitrite was used as an indicator of NO production in the cells. Finally, the absorbance was measured at 540 nm using a OPTIMA microplate reader (FLUOstar, UK). The standard calibration curve was also prepared using according to the kit protocol.

### Phagocytosis activity

The phagocytic ability of macrophage (RAW 264.7) was measured by neutral red uptake assay [36]. RAW 264.7 cells (1 × 10^5^ cells/well) were treated with a series of concentrations of *S. illicilolum* extract (16 - 0.02 μg/μL) in the culture medium at 37 °C and Lipopolysaccharide (LPS) (25 µg/mL) was used as positive control in 96-well plates. After 24 h incubation, the medium was removed, and 50 µL of 0.1% neutral red dissolved in phosphate buffer (PBS) and 50 µL DMEM culture media were added to each well and incubated for 24 h. The cells were washed with PBS three times, and then 100 µL of 1% acetic acid (v/v) in 50% ethanol (v/v) was added to each well to extract the dye phagocytized by macrophages. The absorbance at 540 nm was measured using a OPTIMA microplate reader (FLUOstar, UK).

### In vivo assay

#### Experimental animals

Fifteen, 8-week-old, female New Zealand rabbits weighing 1.6 - 2.2 kg were obtained from the Medical Research Institute in Sri Lanka (MRI). The animals were hold on temperature range 28 ± 2 °C, humidity level 65 ± 5%, and with a 12 h light/dark cycle and fed with a standard pellet diet and water ad-libitum. The study was conducted in accordance with relevant ARRIVE guidelines for reporting animal experiments. The rabbits were acclimatized for 7 days in the experimental site prior to the study. Then, the rabbits were randomly grouped and all the groups were starved for 12 h before the wound formation and seaweed administration. Bedding in the individual cages was covered by a small mesh net, changed daily and cages were kept clean to avoid infection of wounds.

### Experimental design

Rabbits were randomly separated into five experimental groups (n=3) as follows and the treatment was done in all the cases:

*Group I/Treatment I (TI):* Wounds were created and orally treated with 100 mg/kg per day *S. ilicifolium* extract for 14 days.

*Group II/Control (C):* Wounds were created and not given any cream or drug treatment.

*Group III/Treatment II (TII):* Wounds were created and orally treated with 100 mg/kg per day *S. ilicifolium* extract for 21 days (Treatment were started before the induction of skin wounds).

*Group IV/Treatment III (TIII):* Without any wounds and orally treated with 100 mg/kg per day *S. ilicifolium* for 14 days.

*Group V/Normal (N):* Normal Rabbit; kept intact without any treatment and wounds.

The quantity of treatment given to the animals during the experimental time period was calculated with respect to the body weight of animals [37]. Since our previous studies have proven that the CE of *S. ilicifolium* does not possess any cytotoxic effect [35], 100 mg/kg dose was used for the in vivo assay.

### Wound creation

Excision skin wounds (10.40 ± 0.60 mm) were induced in groups I, II, and III. The animals were anesthetized by using ketamine Hydrochloride (ilium Ketamil™ 10% Injection is a dissociative anaesthetic for use singly or in combination with muscle relaxants or tranquilisers 50 mL/Vial, Troy Laboratories Pty Ltd; Australia, 10 mg/kg, IM) and xylazine (ilium Xylazil-100™ 2% is an analgesic, sedative and muscle relaxant injection 50 mL/Vial, Troy Laboratories Pty Ltd; Australia, 2 mg/kg, IM) [38]. The animal was held in standard crouching position, and the mobile skin of flank was gently stretched and held by the fingers. At the first, hair of the test animals’ lower back was completely shaved (60 × 60 mm^2^) and cleared. Following surgery, an outline of the template was traced on the skin using a punch biopsy (10 mm). The four-full thickness circular wounds were made by excising the skin, within the border of the template to the level of loose subcutaneous tissue, using scalpel blade and forceps. Disinfected wounds were washed with sterile saline and povidone-iodine (Win Medicare, India) immediately. All dressings and animal maintenance were in accordance with the ethical rules of standard surgery procedures.

### Size and rate of wound contraction

The size and rate of contraction of wounds were photographed using the digital camera (Nikon Coolpix 4500: Nikon, Tokyo, Japan). Images were analyzed by computerized Carl Zeiss Microscopy GmbH software (Germany) and wound area was measured as the change in pixels. In order to determine the rate of wound healing, animals from both test groups and control groups were held in the standard crouching position. Measurement errors were minimized by repeating each measurement on three times and using an average of the measurements in calculations.

Wound healing percentage in N day = Wound area in the first day - Wound area during the N^th^ day/Wound area in the first day×100

### Histopathological tissue analysis

The tissue samples were collected from the center of the wound and part of the tissue adjacent to the edges of the lesions on 5, 10, 15 and 20 days. The tissue sample from each group was subjected to epidermicrographs (biopsies) was placed in a fixative solution (10 % neutral- buffered formalin), tissue blocks were placed in formalin, dehydrated in a graded series of ethanol, embedded in paraffin, cut into 5 μm thick serial sections. Then the sections were stained with hematoxylin and eosin and examined for the healing process, to identify inflammatory cells, granulation tissue, and tissue structure. H & E grading was performed on the basis of the extent of distance cells migrated from the wound margin and for the analysis of fibroblasts and blood vessels [39]. Van Gieson stain was performed in a differential staining of collagen and other connective tissue and highlight elastic fibers in particular [40].

### Biochemical analysis

Body weights and temperature of all rabbits were recorded once a week, including acclimatization week. Behavioral changes, food and water consumption of rabbit were recorded daily throughout the experimental period. Blood from these groups were collected on 0, 5, 10, 15 and 20 days. The serum enzyme levels of aspartate aminotransferase (AST) and alanine aminotransferase (ALT), and creatinine of serum separated from the collected blood samples [41] using the spectrophotometer (Erba Mannheim, Model: chem- 7, Germany).

### Hematological and serological parameters

Blood smears were prepared to evaluate the white blood cell differential count (WBC-DC) in all groups once a week. The WBC were counted under oil immersion (×100). Furthermore, the following tests were conducted: red blood cells (RBCs) and white blood cells (WBCs), total serum protein (TSP) and packed cell volume (PCV).

### Statistical analysis

All statistical data analysis was performed using Graph Pad Prism Version 9.3.0 (San Diego, CA, USA) software. One-way and two-way analysis of variance was carried out for data analysis whereas Turkey’s method was used for multiple comparisons between the significant levels of interactions of the variables. Each point in the diagrams were shown the mean ± SEM, and differences were considered significant when p<0.05.

## Results

### Composition of the extracts (FTIR and NMR analysis)

**Fig. 1:**
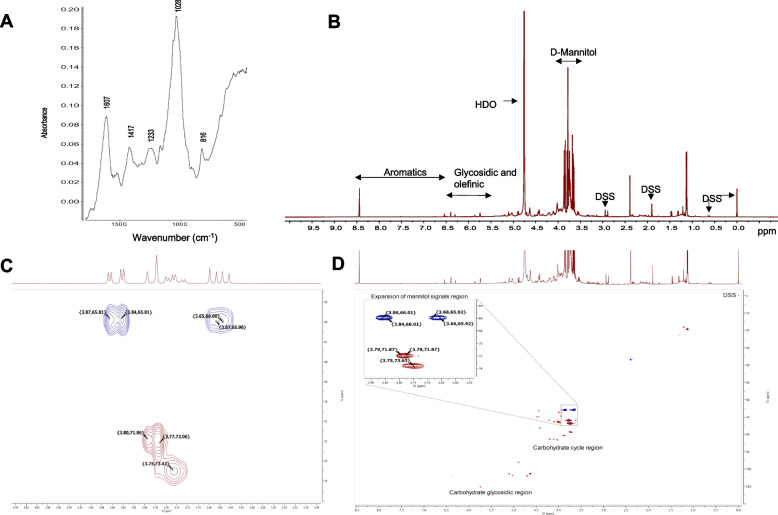
Spectroscopic analysis of *S. ilicifolium* extract. **A**) ATR-FTIR spectrum, **B**) ^1^H-NMR spectrum in D_2_O. D-Mannitol signals between 3.6 and 3.9 ppm. DSS as an internal standard (signal at 0.0, 0.6, 1.9 and 2.9 ppm). 2D HC-HSQC spectra of D-mannitol standard (**C**) and extract (**D**). Expansion in (**D**) shows labeled signals characteristic to D-mannitol

The spectra of the *S. ilicifolium* seaweed revealed different chemical functional groups absorption bands at 1633 - 1650 cm^−1^ (C=C stretching) and 1413 cm^−1^ (O-H bending). The absorption peaks 1234 cm^−1^, 1031 cm^−1^, 886 cm^−1^, 554 cm^−1^, 493 cm^−1^ and 474 cm^−1^ shown the existence of C-O stretching band (Fig. 1A). Moreover, strong peaks in the range of 1628 to 1428 cm^−1^ entitled asymmetric and symmetric stretching vibrations that certified to carboxylate anions (COO−) [42]. In addition, it has been showed the strong IR absorption bands at 1602 cm^-1^ are mainly due to C=C stretching in α, β-unsaturated ketone. ^1^H NMR spectroscopy is the main technique used in the analysis of chemical composition and structural patterns [43]. Figure 1B shows the ^1^H NMR spectrum of *S. ilicifolium* extracts. Spectrum of standard sample of D-Mannitol is given in a Fig. 1 CD. Spectrum shows characteristic signals of the D-mannitol in *S. ilicifolium* extracts in a region of 3.5-3.9 ppm (Fig. 1). The chemical composition of *S. ilicifolium* CE indicated the mannitol as a major bioactive compound, which, according to literature, actively participates in the wound healing process in injured tissues by increasing the viability of collagen fibrils and preventing further cell damage [44]. Moreover, mannitol is able to modulate wound cell function by enhancing the proliferation and migration of fibroblast cells [45, 46].

### In vitro assay

#### Cytotoxicity effects

**Fig. 2:**
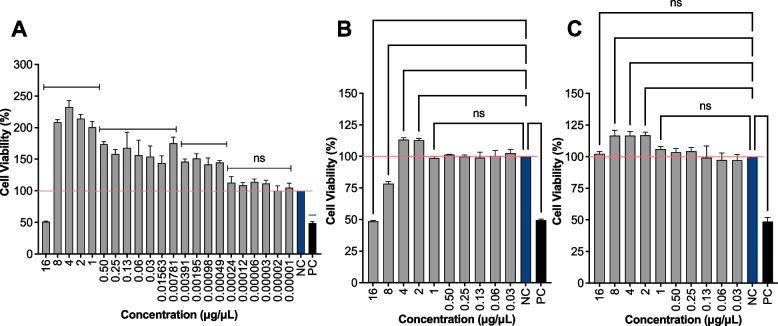
The cell viability ability of **A)** RAW 264.7 (macrophages), **B)** Human Dermal Fibroblasts (HDF), **C)** Human Immortalized Keratinocyte (HaCaT) cells were treated with *S. ilicifolium* (16 to 0.02 μg/μL). Values are expressed as mean ± SE; Data is compared against values in the control group. (*) indicates statistically significant difference from respective group using ANOVA, followed by Tukey comparisons test (*p* > 0.05). (ns) indicates statistically no significant difference from control group using ANOVA, followed by Tukey comparisons test (*p* > 0.05). MilliQ water was used as negative control (NC-without treatment)

This study is the first to highlight the non-toxic effect of *S. ilicifolium* on RAW 264.7, HDF and HaCaT cells. A range from 8 - 16 μg/μL CE extracts displayed cytotoxic effects on the HDF cell line (Fig. 2). Accordingly, a remarkable difference in cell viability of cell lines against to the concentration gradient of 8 -16 μg/μL noted. Our previous In vitro experimentation on L929 proposed that *S. ilicifolium* extract was non-toxic to L929 cells [28].

### Cell proliferation

**Fig. 3:**
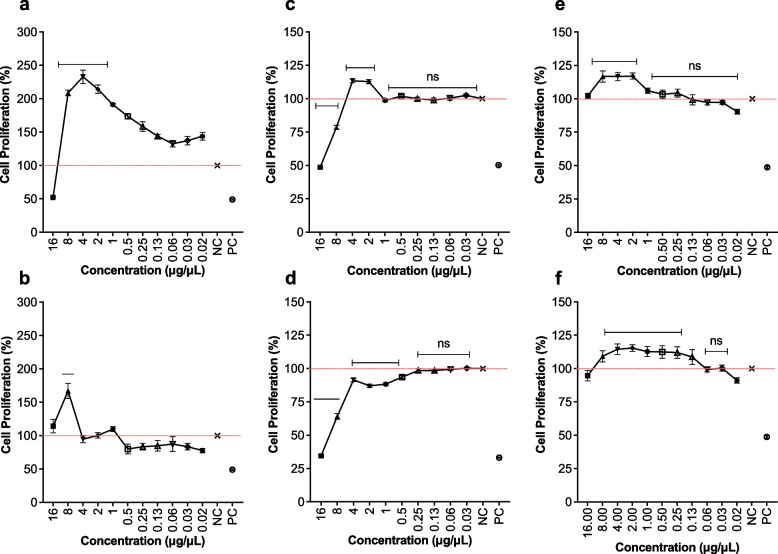
The proliferation of RAW 264.7 (macrophages) (**a**-24h, **b**-48h), Human Dermal Fibroblasts (HDF) (**c**-24h, **d**-48h), Human Immortalized Keratinocyte (HaCaT) (**e**-24h, **f**-48h) cells treated with *S. ilicifolium* (16 to 0.02 μg/μL) and negative control were evaluated over the course of 24 and 48 h. Cell proliferation is given as a percentage of negative control cells. The given values are expressed as mean ± SEM (n=8). (*) indicates statistically significant difference and (ns) indicates statistically no significant difference from the control group using ANOVA, followed by Turkey multiple comparisons test (P>0.05)

AlamarBlue assay, exhibited a vast range of ability of *S. ilicifolium* extract to induce of cell proliferation and/or migration without any Cytotoxicity effects on RAW 264.7, HDF and HaCaT cell lines (Fig. 3). *S. ilicifolium* extract concentration of 2 - 4 μg/μL was able to exhibit the quickest cell proliferation when they added to RAW 264.7 cells. The cell proliferation acceleration lasts up to 48 h, and then gradually reduced the speed of cell proliferation (Fig. 3 a,b). It indicates that there is an optimum concentration of *S. ilicifolium* extract to induce cell proliferation. The CE has showed gradual increase in the speed of HDF cell proliferation with concentration gradient from 16 - 4 μg/μL in initial 24 h. However, concentrations ranged from 8 - 16 μg/μL indicated to be toxic on cells in 24 - 48 h of post treatment of *S. ilicifolium* extracts (Fig. 3 c,d). *S. ilicifolium* extracts concentrations from 2 - 8μg/μL was showed the highest cell proliferation and/or migration activities on HaCaT cells compared to all other concentrations (Fig. 3 e,f).

### Cell migration

**Fig. 4:**
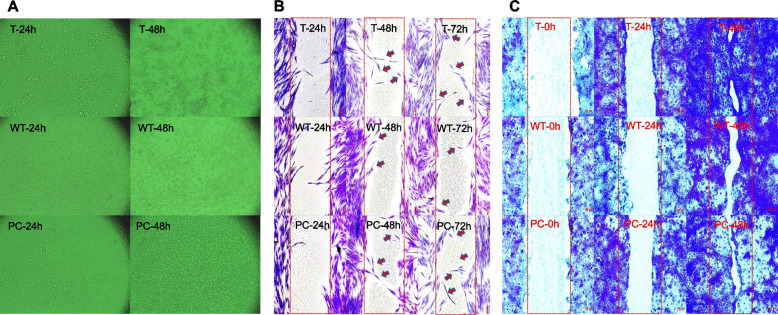
**A)** Microscopic inspection (40 × magnification) of RAW 264.7 (macrophages) cells, **B)** Human Dermal Fibroblasts (HDF) cell migration, **C)** Human Immortalized Keratinocyte (HaCaT) cell migration treated with *S. ilicifolium* (4 μg/μL) and negative control were evaluated over the course of 24 and 48 h. Cell migration observed after injury to the cellular monolayer from the in vitro scratch wound healing assay. Microscopic inspection (10× magnifications) of immediately after scratching (0 h) and after 24 and 48 h of wound healing. WT- control/without treatment, PC-positive control/Lipopolysaccharide (100 μg/mL), T- Treatment *S. ilicifolium* (4 μg/μL) aqueous extracts treated cells. Scale bar is 100 μm. Values are expressed as mean ± SE; Data is compared against values in the control group

**Fig. 5:**
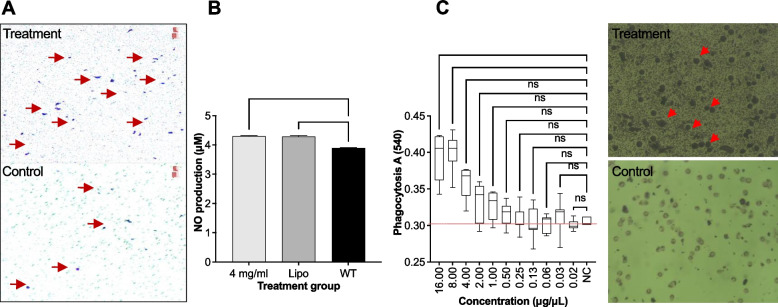
**A)** Transwell assay; Microscopic inspection (40× magnification) of RAW 264.7 cells after 24 h incubation with *S. ilicifolium* extracts (4 μg/μL), **B)** Level of nitric oxide (NO) production when threated with *S. illicilolum* extract (4 μg/μL), **C)** phagocytosis ability in RAW 264.7 cells. Positive control- Lipopolysaccharide (25 μg/mL). Microscopic inspection (10× magnifications) before and after neutral red uptake during 24 h of phagocytosis ability. (Red arrow shows neutral red partial uptake). The given values are expressed as mean ± SEM (*n*=8). (*) indicates statistically significant difference and (ns) indicates statistically no significant difference from the control group using ANOVA, followed by Turkey multiple comparisons test (*P*>0.05)

The present study represented the second phase of wound healing described by the proliferation and migration of human epidermal keratinocyte (HaCaT). In general, it takes 48 h to close the gap in HaCaT (keratinocytes) monolayer cells of Epithelial cell line [47]. Further, a previous study provides the evidences of ability of aqueous extract of seaweeds in promoting cell proliferation and migration activities on the L929 cells in the scratch wound healing assay [35]. Analysis of images revealed that (2 μg/μL) is the optimum concentration that need to close the wound gap in the confluent cell monolayer (Fig. 4). Accordingly, this concentration was able to exhibit a significant statistical relationship with control group (*p*<0.05). Mobilization of keratinocytes and closer the gap was taken place in 24 h time in the presence of *S. ilicifolium* extracts and it was statistically significant (*p*<0.05) Moreover, these statistical relationships were able to exhibit potential effects of indicated that the aqueous extract of *S. ilicifolium* on wound healing process. Transwell assay has been widely used for studying the motility of different types of cells including immune cells and cancer cells [48]. Cell migration through the membrane was represent that extract compounds act as chemoattractants. In order to determine the best concentration; 4 μg/μL has showed the highest cell migration (Fig. 5A) activity compared to the control group.

### Nitric oxide level

As can be seen from the Fig. 5B, S*. ilicifolium* extracts significantly stimulated the production of NO in RAW 264.7 cells at 4 μg/μL concentration when compared to control group.

#### Phagocytosis activity

Macrophages have a defensive function against pathogens such as microbes, and play an important role in the homeostatic maintenance of the body through the disposal of internal waste materials and tissue repair [49]. Macrophages and dendritic cells play a key role in the innate immune system by recognizing and removing bacteria [49]. Phagocytic activities of RAW 264.7 cells treated by *S. ilicifolium* extracts were evaluated by the uptake of neutral remand compared with the control group noted that extract concentrations ranged 4 -16 μg/μL enhanced significantly (Fig. 5C), (*p*<0.05). It suggested influence of *S. ilicifolium* extracts on wound healing process.

### In vivo assay

#### Wound healing

**Fig. 6:**
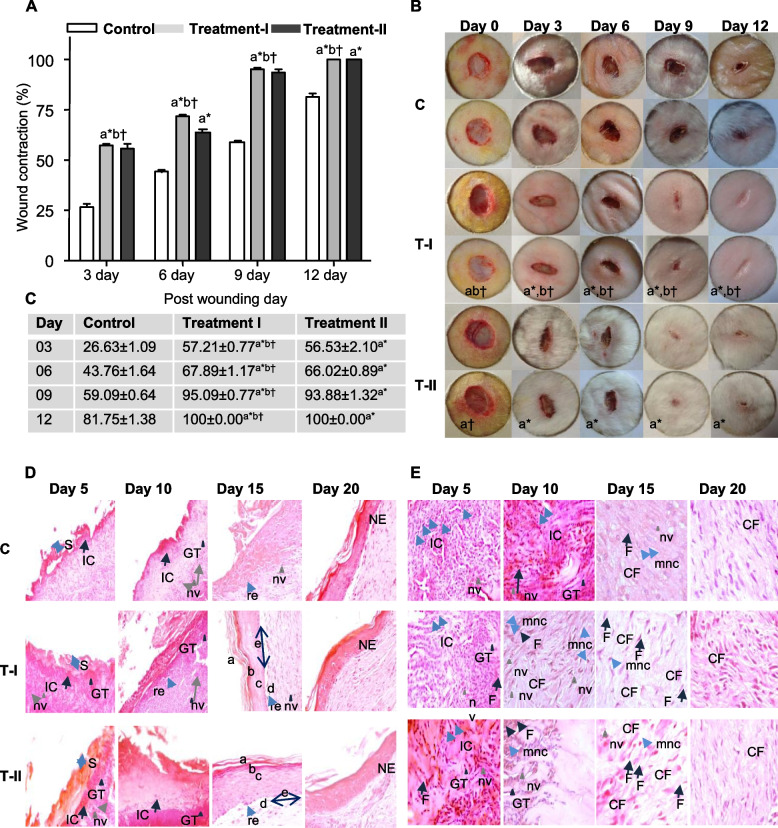
Percentage of wound contraction and digital photograph of wounds. (**A**) The average healing time and percentage of wound healing in each group, (**B**) A digital photograph of the progressive wound healing activity of excision wound model in each group, (**C**) Wound contraction (%) on different days. The given values are the diameter of wounds expressed as mean ± SEM (n=12). a = denote when compare to the control group, b = denote when compare to the treatment II Group, (*) indicates statistically significant difference and (†) indicates statistically no significant difference from the respective group using ANOVA, followed by Turkey multiple comparisons test (P>0.05). **D** and **E**). Histological photomicrograph of hematoxylin and eosin stained sections of wound tissues. (**D**). Epidermis photomicrographs of sections (H and E stain; ×40). Skin appears well-rearranged epidermis. (a) stratum corneum, (b) stratum granulosum (c) stratum spinosum, (d) stratum basale, (e) papillary layer. (**E**). Demonstrating granulation tissues (H and E stain; ×100). Arrows pointing the events during wound healing, s: scab, re: re-epithelialization, GT: granulation tissue, nv: neovascularization, IC: inflammatory cells, mnc: mononuclear, CF: collagen fiber, F: fibroblasts, NE: new epithelium. C (Control): received an equal amount of distilled water, orally, Treatment I (T-I): received *S. ilicifolium* CE (100 mg/Kg BW/day for 14 days, orally), Treatment II (T-II): received *S. ilicifolium* CE (100 mg/kg BW/day for 21 days, orally)

Comparison between animals treated with seaweeds extracts and non-treated control animals have shown a significant difference. The wounds of wounded rabbit group treated with *S. ilicifolium* CE, were completely healed within 12 days (Fig. 6A). The rates of wound contraction were expressed as the wound area (mm^2^) on a day that had healed (Fig. 6B).

In comparison to group II (26.63 ± 1.09%), highest wound healing was observed in group I (57.21±0.77%), on day 3. On day 9, wound contractions were 95.09 ± 0.77%, 59.09 ± 0.64%, and 93.88 ± 1.32% for groups I, II, and III, respectively. The area of the wounds on the first day was considered as 0% and the wound areas on subsequent days were compared with the control wound on every 03, 06, 09 and 12 days (Fig. 6). Healing percentages on different days of treatments were calculated as the difference between the initial wound surface area and that on the day of measurement. Treatment I and III group trend of healing in these groups was very similar without any significant differences (Fig. 6C).

The rates of wound healing were affected by the morphological evolution during the repair process, where the most important event was re-epithelialization. The re-epithelialization surface and at day 10, the area of the re-epithelialized surface at the wound center was greater in the *S. ilicifolium* CE treated group when compared with the control (Fig. 6D). At days 10 and 15, the epidermal layer was extent than the Control. At day 10 to 15, the re-epithelialized surface having a peak in the Control. The proliferative phase was initiated with re-epithelialization involving the extracellular matrix (ECM) and collagen production [50]. At day 5, the *S. ilicifolium* CE have shown high migration and proliferation of keratinocytes and fibroblast cells. At day 10, the *S. ilicifolium* CE has shown migration inhibited and proliferation stimulated. In the Control, the keratinocytes and fibroblast cells migration were observed at days 10, and fibroblast cells proliferation at days 15. The results indicated *S. ilicifolium* CE accelerated re-epithelialization compared to the Control. The re-epithelialization area (after 5 days) and for epidermal thickness (days 10 and 15) were always higher in the *S. ilicifolium* CE.

Granulation tissue was consisting of fibroblasts (F), collagen fibers (CF), and new vessels (nv). The Fibroblasts were responsible for producing collagen and proliferation of this cell type [50, 51]. At this stage of wound healing during days 5 to 10 has been shown *S. ilicifolium* CE treated rabbit group. The dermis at days 15 was visible better-organized collagen fibers than in the Control (Fig. 6). During the fibroblast migration, proliferation and maturation, the process of wound contraction extend its highest efficiency. The rate of the re-epithelialized, tissue granulation and mature collagen fibers has been shown a higher proliferative activity in wounds when treated with the *S. ilicifolium* CE. Even with the remodeling of the number of new blood vessels (nv) and the proliferation and differentiation of keratinocytes and fibroblast cells, has increased until 10 days of the treatment group. At 15 days, the wound contraction, Skin appears well-rearranged epidermis., and formation of collagen fibers have shown enhanced the *S. ilicifolium* crude extracts treatment group when compared with the control group (Fig. 6).

### Toxic effects

#### Body weight and body temperature

**Fig. 7:**
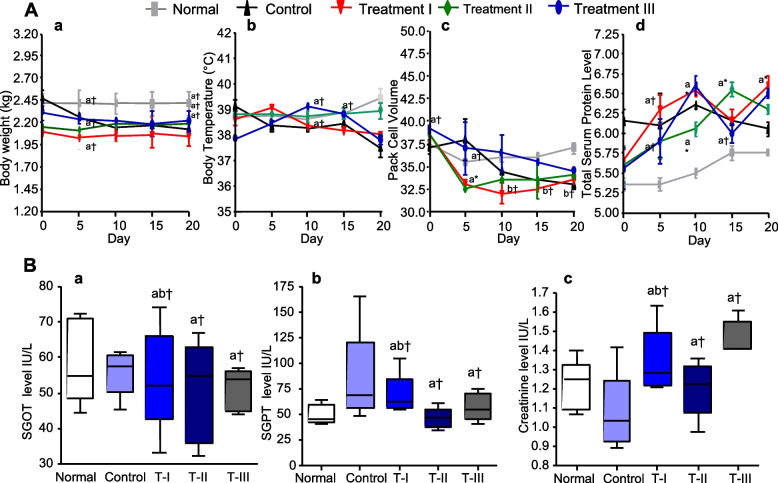
Body weight, temperature, hematological and serological parameters. (**A**- a). Weights of rabbit and effects of toxicity before and after treatment, b). Body temperature of rabbit in each experimental groups, c). Pack cell volume d) Total serum protein level. Values are expressed as mean ± SEM. a = denote significant different of the body weight from day 0, b = denote significant different of body weight from day 20. **B-** a). AST levels in rabbit, (B). alanine aminotransferase (ALT) levels in rabbit, (**C**). Serum creatinine levels in the rabbit. Data are presented as the mean ± SEM of n=04 rabbit of each experimental group; Data is compared against the normal group. a = denote when compare to the control group, b = denote when compare to the treatment II Group, (*) indicates statistically significant difference and (†) indicates statistically no significant difference from the respective group using ANOVA, followed by Tukey multiple comparisons test (P>0.05). Control: received an equal amount of distilled water, orally, Treatment I (T-I): received *S. ilicifolium* CE (100 mg/Kg BW/day for 14 days, orally), Treatment II (T-II): received *S. ilicifolium* CE (100 mg/kg BW/day for 21 days, orally), Treatment III (T-III): received *S. ilicifolium* CE without wound (100 mg/kg BW/day for 14 days, orally)

Any significant change of body weight (Fig. 7A), temperature (Fig. 7b) and behavioral changes in the rabbit of both experiment and control groups were not reported during the experimental period. Nevertheless, there was no any significant difference of average body weight between treatment groups (I, II and III) and control rabbit groups (wounded and non-wounded). These results shown that the body weights (kg) of rabbits have not been affected due to oral administration of aqueous extract of *S. ilicifolium* throughout the experimental period. Further, dietary habits (food and water intakes) of the control and treatment groups were not changed significantly during the experimental period (Fig. 7).

### Biochemical analysis

AST, ALT and serum creatinine levels in blood collected from all the groups of rabbits are represented in Fig. 7B. It is evident that the values AST, ALT and serum creatinine levels of blood serum samples obtained from all the groups of rabbits have not exhibited a significant difference from their normal ranges; SGOT/AST: 14-113 IU/L, SGPT/ALT: 12- 67 IU/L, Serum Creatinine: 0.8-1.8 mg/dl (Laboratory Rabbit, research animal resources, University of Minnesota). Hence, AST, ALT and serum creatinine indicated that there were no toxic effects in seaweed treated rabbit groups (Table 1).

**Table 1:** Biochemical parameters of rabbit of each test group over a period of 20 days.

**Biochemical test**	**Normal**	**Control**	**Treatment I**	**Treatment II**	**Treatment III**
AST (IU/L)	51.77±9.96	46.86±9.52	45.86±10.66^ab†^	42.41±9.31	42.18±8.30^a†^
ALT (IU/L)	44.89±4.80	76.66±24.85	60.55±14.16^ab†^	39.07±8.22	47.34±11.23^ab†^
Creatinine (mg/dl)	1.216±0.06	1.072±0.091	1.34±0.08	1.20±0.064	1.47±0.04

Values are expressed as mean ± SEM; Data is compared against a control group. *a* = denote when compare to the control group, *b* = denote when compare to the treatment II Group, (*) indicates statistically significant difference and (†) indicates statistically no significant difference from respective group using ANOVA, followed by Tukey comparisons test (P > 0.05). Control: received an equal amount of distilled water, orally, Treatment I: received *S. ilicifolium* CE (100 mg/Kg BW/day for 14 days, orally), Treatment II: received *S. ilicifolium* CE (100 mg/kg/BW/day for 21 days, orally), Treatment III: received *S*. *ilicifolium* CE without wound (100 mg/kg BW/day for 14 days, orally)

### Hematological and Serological parameters

Rabbit groups treated with seaweeds CE of *S. ilicifolium* (100 mg/kg) did not exhibit any significant effect in PCV, TSP in comparison with compared to the control group (Table 2).

**Table 2:** Packed cell volume and serum protein levels (SPL) on the experiment period.

	**Normal**	**Control**	**Treatment I**	**Treatment II**	**Treatment III**
**PCV**	37.20±1.10	35.90±1.44	34.90±2.16^ab†^	34.40±1.05^a†^	37.40±1.58^a†^
**SPL**	5.43±0.17	6.16±0.05	6.25±0.17 ^ab†^	6.080±0.16^a†^	6.11±0.19^a†^

Values are expressed as mean ± SEM; Data is compared against a control group. *a* = denote when compare to the control group, *b* = denote when compare to the treatment II Group, (*) indicates statistically significant difference and (†) indicates statistically no significant difference from respective group using ANOVA, followed by Tukey comparisons test (*P* > 0.05). Control: received an equal amount of distilled water, orally, Treatment I: received *S. ilicifolium* CE (100 mg/Kg BW/day for 14 days, orally), Treatment II: received *S. ilicifolium* CE (100 mg/kg/BW/day for 21 days, orally), Treatment III: received *S*. *ilicifolium* CE without wound (100 mg/kg BW/day for 14 days, orally)

Oral treatment showed increased red blood cells count during the 0 to 10 days of treatment (Table 3), however, there was an indicated that prolonged treatment for up to 15 days increased the red blood cells count. Rabbit showed that the oral treatment of seaweeds extracts of 100 mg/kg increased the white blood cells count during the 5 days and it has shown to decrease during 5 to 10 days. However, it has been shown reasonably increased the red blood cells count thus increasing the oxygen supply to the wound site [52] as seen in the healing of rabbit.

**Table 3:** WBC and RBC count during the experimental period.

**Test**	**Day**	**Normal**	**Control**	**Treatment I**	**Treatment II**	**Treatment III**
RBC	0	3.32± 0.18	3.08± 0.11	3.75± 0.11	4.34± 0.25	3.79± 0.11
	5	4.80±0.10	5.25±0.03	5.47± 0.07	3.96± 2.03 ^a†^	5.15± 0.48
	10	5.60± 0.02	4.69± 0.38	8.20±0.39	7.99± 0.37 ^a*^	7.24± 0.07
	15	5.66± 0.29	7.19± 0.04	7.80±0.14	10.97± 1.40 ^a*^	7.63± 0.28
	20	5.89± 0.29	7.23± 0.07	9.21± 1.05	8.16± 0.08	7.44± 0.38
WBC	0	7.95±0.20	7.85±0.55	8.70±0.26	7.82±0.82	6.45±0.26
	5	6.47±0.04	9.87±0.27	7.55±1.07	9.37±0.73	6.90±1.04
	10	6.32±0.79	7.40±0.32	8.87±0.62	6.60±0.14	11.03±2.06
	15	6.97±0.01	10.15±1.12	7.20±0.66	7.87±0.27	8.50±0.03
	20	7.77±0.27	7.30±0.49	7.72±1.54	4.92±0.13	7.62±0.24

Values are expressed as mean ± SEM; Data is compared against a control group. *a* = denote when compare to the control group, *b* = denote when compare to the treatment II Group, (*) indicates statistically significant difference and (†) indicates statistically no significant difference from respective group using ANOVA, followed by Tukey comparisons test (*P* > 0.05). Control: received an equal amount of distilled water, orally, Treatment I: received *S. ilicifolium* CE (100 mg/Kg BW/day for 14 days, orally), Treatment II: received *S. ilicifolium* CE (100 mg/kg/BW/day for 21 days, orally), Treatment III: received *S. ilicifolium* CE without wound (100 mg/kg BW/day for 14 days, orally)

## Discussion

Natural products including terrestrial and marine flora are rich in secondary metabolites that have unique structural orientations, which are easily malleable for drug development [53]. The main categories of secondary metabolites derived from natural products and that have been developed into drugs are: terpenes (34%), glycosides (32%), polyketides and others (18 %) and alkaloids (16%) [40]. An analysis of the origin of the drugs developed between 1981 and 2001 showed that 80% of 122 plant-derived drugs were discovered as a result of chemical studies directed at isolating the biologically active substances from the natural products used in traditional medicines [54]. Between 2000 and 2005, about five medicinal natural products-based drugs including antimicrobial drugs were introduced in the United States market and another seven natural products-derived compounds are currently in clinical trials around the world [55, 56]. Despite many natural products especially medicinal plants are used in traditional medicine for treating wounds, not much of them have been clinically tested as wound healing agents. Wound healing is a dynamic process involving a complex interplay of various cells, extracellular matrices, and soluble mediators [57, 58]. The wound healing immediately begins after an injury to a smooth interaction among different types of tissues and cells [59] and specific process leading to the restoration of injured tissues [9]. This is followed by attraction and proliferation of fibroblast, which is the connective tissue cell responsible for collagen deposition that is needed to repair the tissue injury [60]. Cell migration or proliferation is known to be involved in skin regeneration, granulation and wound healing [61]. Recently, our laboratory developed an in vivo rabbit model to investigate a healing process and understand the wound healing ability of orally treated seaweed extracts. Initial findings observing the wound healing activity showed a reduced healing time. In this study, the *S. ilicifolium* has significantly enhanced wound healing in rabbits and reduced the days needed for complete healing compared with the non-treated control groups. This could be due to an effect of several groups present in the seaweed extracts which may enhance some stage in the wound healing process. The wound healing activity of *S. ilicifolium* treated rabbits has demonstrated significant healing effects when compared to the control group (P<0.05).

According to the histopathological findings, it is clear that the wound healing activity was significantly faster in the rabbit group (treatment I and II) treated orally with *S. ilicifolium* CE than other wounded rabbit groups (treatment III and control). Enhanced wound healing activity could be attributed to increased collagen formation and angiogenesis [32, 62]. Oral administration is non-painful, safest route and has immediately responded for the wound healing process. Tissue granulation in the wounded sites was significantly increased in the histopathological sections of the animals treated with *S. ilicifolium* CE compared to the control. Furthermore, this group has exhibited an increased rate of epithelialization and wound contraction of wound. Wound contraction was evaluated by analyzing the change in diameter of the wound, to differentiate it from re-epithelialization. This study highlighted that re-epithelialization plays one of the major roles in wound healing activity when treated with *S. ilicifolium* CE. It is worthwhile in future to determine the bioactive compound(s) of *S. ilicifolium* or seaweeds.

Previous studies on its close relative - brown algae - showed that their main components are polysaccharides, which mainly consist of fucoidan, laminarin, cellulose, alginates, mannitol, algal fucans, galatians and alginates [63]. Polysaccharides such as fucoidans are reported to have an effect on the traditional medicine for immunomodulatory and inflammatory [64]. Natural products in general has been known to possess a strong potential for the treatment of skin wounds [53, 65, 66]. The presence of complex halogenated and non-halogenated terpenoids [67] in seaweeds may be responsible for their wound healing capacity. Since mannitol are reported to improve wound healing and protect tissues from oxidative damage [68], it is likely that the mannitol-rich *S. ilicifolium* extract is exerting the wound healing activity observed in this study.

This study demonstrated that an oral administration of *S. ilicifolium* CE enhanced cutaneous healing of wound within first 12 days. *S. ilicifolium* CE (100 mg/kg) did not show any significant toxicity effect on rabbits. Further, any significant effect on serum protein level and pack cell volume was also not reported compared to the wounded control group. Generally, body weight is considered as a sensitive indicator of experimental animals and the change in body weight is used to estimate the toxic effects of drugs for animals in toxicological studies [69, 70]. Toxicity effect can be investigated on liver cells, which get damaged as a result of the introduction of infectious agents or chemicals, and as a result the serum levels of ALT and AST tend to increase significantly [41, 71]. This was not observed in the present study. The serum enzymes such as ALT, AST, and creatinine were found to be within the normal range in the seaweed extract (*S.ilicifolum*) treated groups (Group I, II and III) and without treatment of the control groups (Group IV and V). The toxicity result indicates that *S. ilicifolum* seaweed extract is safe for human consumption.

According to the findings, *S. ilicifolium* CE can be used as an immunostimulant for analysing macrophage-related inflammatory responses. Furthermore, these crude extractions may also be clinically useful in modifying macrophage function in diseases where it is impaired or needs to be enhanced. Due to the central role macrophages play in both innate and acquired immune responses, we investigated whether the combination of D-Mannitol and aromatic compounds obtained from *S. ilicifolium* could modify the activity of RAW 264.7 murine macrophages in vitro. *S. ilicifolium* CE can be produced by activated macrophages during inflammatory responses. The presence of NO in the body suggests that it is necessary for maintaining health. One of the most significant molecules for blood vessel health is NO, produced by nearly all types of cells in the human body [72, 73]. In the present study, *S. ilicifolium* CE stimulated the release of nitric oxide from RAW 264.7 macrophages. Inflammatory responses are regulated by NO, which has a wide range of biological activities. As well as enhancing physical performance and reducing muscle soreness, nitric oxide helps manage type 2 diabetes and erectile dysfunction [74].

Wounds - particularly chronic wounds - are the major concerns for the patient and clinician alike. Research on wound healing agents is one of the developing areas in modern biomedical sciences. The seaweeds are used as medicines, especially in the Asian countries, where modern health services are limited [28]. However, the mechanism of wound healing process remains unknown. In this study, oral administration of *S. ilicifolum* has enhanced the rate of wound healing increase in collagen synthesis and tensile strength of the wound tissues. As we know in a moist environment, exudate provides the cells involved in wound repair with nutrients, controls infections, and provides the best environment for healing [75]. Developing countries have to spend a large sum of money on importing drugs and machines used in wound healing treatment. Finding alternative, effective and less expensive methods for treatments of wound healing would bring immense benefits.

## Conclusion

*S. ilicifolum* extract significantly aided the healing process of wounds. Histopathological findings has revealed that the healing process of wounds formed in rabbits treated orally with CE of *S. ilicifolium* was significantly faster in the group treated orally with, which enhanced tissue granulation and epithelization compared to the control group. The mechanism of action remains unknown. It is likely that the wound healing have occurred possibly by the promotion of fibroblast proliferation, which results in higher rates of wound closure. Further investigation of cellular responses to the experimentally induced wound formation in the rabbit model may help to clarify the role of wound repair. This interesting finding could help us consider *S. ilicifolum* as an ideal wound healing agent especially in Asian countries where the modern hospital treatments are expensive and not readily accessible. However, a clear understanding of the safety of long-term ingestion of seaweeds in an animal model/human clinical trials would be vital for engaging the extracts for the treatment of wounds. Alternatively, the extracts can be loaded in the band aid to fasten wound healing process or the CE could be also made as lotions/ointments for treating chronic wounds. Since pure compounds are easier for drug development and preparing effective doses, isolation of pure active principal compounds from this CE and development of novel wound healing drugs would be worthwhile to pursue in near future.

## Data Availability

The datasets and materials are contained during the in this study. Animals were obtained from the Medical Research Institute (MRI) Colombo Sri Lanka.

## Abbreviations

*S. ilicifolium*: *Sargassum ilicifolium*
BW: Body Weight
CE: Crude Extracts
RT: Room Temperature
^°^C: Celsius
AST: Aspartate aminotransferase
ALT: Alanine aminotransferase
SEM: Stranded Error of the Mean
