# Correction: A novel therapeutic effect of mannitol-rich extract from the brown seaweed *Sargassum ilicifolium* using in vitro and in vivo models

**DOI:** 10.1186/s12906-023-03879-z

**Published:** 2023-02-15

**Authors:** Amal D. Premarathna, Rando Tuvikene, MNR Somasiri, MLWP De Silva, Ranjith Adhikari, TH Ranahewa, RRMKK Wijesundara, SK Wijesekera, IPGHU Dissanayake, Phurpa Wangchuk, Vitalijs Rjabovs, Anura P. Jayasooriya, RPVJ Rajapakse

**Affiliations:** 1 School of Natural Sciences and Health, Tallinn University, Narva mnt 29, 10120 Tallinn, Estonia; 2 Department of Veterinary Pathobiology, Faculty of Veterinary Medicine and Animal Science, University of Peradeniya, Peradeniya, Sri Lanka; 3 South Asian Clinical Toxicology Research Collaboration. Faculty of Medicine, National Serpentarium, University of Peradeniya, Peradeniya, Sri Lanka; 4 Department of Zoology, Faculty of Natural Sciences, Open University, Kandy Regional Center, Polgolla, Sri Lanka; 5 Department of Basic Veterinary Sciences, Faculty of Veterinary Medicine and Animal Science, University of Peradeniya, Peradeniya, Sri Lanka; 6 Centre for Molecular Therapeutics, Australian Institute of Tropical health and Medicine, James Cook University, Smithfield, QLD 4878 Australia; 7 National Institute of Chemical Physics and Biophysics, Akadeemia tee 23, 12618 Tallinn, Estonia

**Correction: BMC Complement Med Ther 23, 26 (2023)**

**https://doi.org/10.1186/s12906-023-03840-0**

Following publication of the original article [1], the author reported that the first author's affiliation 1 will be "School of Natural Sciences and Health, Tallinn University, Narva mnt 29, 10120 Tallinn, Estonia. Affiliations to authors have been updated and are presented correctly in this correction article.

The original article [1] has been corrected.
